# New stereolithographic resin providing functional surfaces for biocompatible three-dimensional printing

**DOI:** 10.1177/2041731417744485

**Published:** 2017-12-21

**Authors:** Andreas Hoffmann, Holger Leonards, Nora Tobies, Ludwig Pongratz, Klaus Kreuels, Franziska Kreimendahl, Christian Apel, Martin Wehner, Nadine Nottrodt

**Affiliations:** 1 Fraunhofer Institute for Lasertechnology, Aachen, Germany; 2 Department of Biohybrid & Medical Textiles, Institute of Applied Medical Engineering, RWTH Aachen University, Aachen, Germany

**Keywords:** Bioprinting, functional surface, stereolithography, thiol-ene, photochemistry

## Abstract

Stereolithography is one of the most promising technologies for the production of tailored implants. Within this study, we show the results of a new resin formulation for three-dimensional printing which is also useful for subsequent surface functionalization. The class of materials is based on monomers containing either thiol or alkene groups. By irradiation of the monomers at a wavelength of 266 nm, we demonstrated an initiator-free stereolithographic process based on thiol-ene click chemistry. Specimens made from this material have successfully been tested for biocompatibility. Using Fourier-transform infrared spectrometry and fluorescent staining, we are able to show that off-stoichiometric amounts of functional groups in the monomers allow us to produce scaffolds with functional surfaces. We established a new protocol to demonstrate the opportunity to functionalize the surface by copper-catalyzed azide-alkyne cycloaddition chemistry. Finally, we demonstrate a three-dimensional bioprinting concept for the production of potentially biocompatible polymers with thiol-functionalized surfaces usable for subsequent functionalization.

## Introduction

Tissue engineering is currently one of the fields of interest in regenerative medicine. Currently, building up whole organs is one of the most sophisticated tasks in the area of tissue engineering and could be one approach to address the needs of our aging society. One important step on the road toward artificial organs is the development of organ-on-a-chip systems which can mimic organs and the interplay of those. Those systems have already been developed.^1^ Nevertheless, developing whole organs, for example, to replace a heart or just a blood vessel, actually suffers from missing scaffolds which provide the mechanical and geometrical properties to support cell growth and tissue maturation.^2^ Nowadays, three-dimensional (3D) bioprinting is one of the most promising technologies for the tailored production of scaffolds. Several rapid prototyping technologies used for 3D bioprinting, for example, fused deposition modeling,^3^ laser-assisted bioprinting,^4^ or stereolithography^2^ show their benefit in implant printing, because they allow for controlled geometries and adapted mechanical properties.^5^ Besides process controlling, the material development itself is important to get materials fulfilling all the demanding requirements of tissues and organs, for example, being biocompatible and providing the right mechanical properties.^6,7^ In this work, we investigated a class of polymeric materials useful for laser-based stereolithographic processes. The used monomers comprise two classes of monomers containing at least two alkene or thiol groups. Those two components react spontaneously under ultraviolet (UV)-irradiation at a wavelength of approximately 266 nm. The observed reaction is also known as thiol-ene-click reaction fulfilling all the requirements of such a reaction.^8^ Because thiol groups are activated directly by UV-light (λ < 300 nm), there is no need for a photoinitiator in this reaction. Photoinitiators often cause problems concerning the biocompatibility of a polymer and are responsible for fast aging.^9,10^ The benefit of this class of materials can be underpinned by studies of other groups. Caldwell et al.^11^ used this chemistry to create biocompatible sponge like structures. In a study of Barker et al., side-chain-functionalized poly(carbonate) was crosslinked via thiol-ene chemistry within a microstereolithographic process. In this study, good biocompatibility was demonstrated even though a photoinitiator (Irgacure 784) was used to crosslink the material with light at λ = 465 nm.^12^ In a recently published study, Stichler et al.^13^ developed thiol-ene-based hydrogels based on poly(glycidol) which seem to be useful for 3D printing of bone marrow–derived mesenchymal stem cells.

Beside the opportunity to print without photoinitiators, this class of material can be used as off-stoichiometric thiol-ene resins. This means that either thiol or alkene functionalization at the surface can be achieved, which might be useful for further surface functionalization.^14^

Within this work, we investigated the UV light–induced polymerization of pentaerythritol tetrakis(3-mercaptopropionate) and poly(ethylene glycol) divinyl ether for 3D printing of scaffold-like structures. We used two functional group ratios and tested the resulting polymers for biocompatibility. The off-stoichiometric material combination was chosen in a ratio of 2:1 to achieve high amounts of surface functional groups designed for a subsequent functionalization. We successfully demonstrated the activity of these functional groups by light-induced addition of a linker molecule and subsequent catalytic addition of a fluorescence dye.

## Materials and methods

### Preparation of thiol-ene flat specimens

Flat specimens of thiol-ene polymer were fabricated by polymerization of a resin consisting of the alkene-monomer poly(ethylene glycol) divinyl ether (Mw = 250 g mol^−1^; Sigma Aldrich Chemie GmbH, Taufkirchen, Deutschland) and the thiol-monomer pentaerythritol tetrakis(3-mercaptopropionate; Sigma Aldrich Chemie GmbH) in functional group ratios of 1:1 and 2:1 as described in Table 1.

**Table 1.** Thiol-ene photo resin formulation.

Resin	Tiol–alkene ratio	m(thiol) (g)	m(alkene) (g)	V (resin) (mL)
A	1	1.12	1.15	2
B	2	1.56	0.80	2

The monomers were mixed using a vortex mixer (VWR International GmbH, Langenfeld, Germany). Resin-coated (75 µL) microscopy slides were placed in a distance of 3 cm under a UV lamp (UVP 3UV-38, 8W; Thermo Fischer Scientific, Waltham, MA, USA). Curing was achieved at a wavelength of λ = 254 nm, a power of P = 2.6 mW, and t = 10 min.

Phase separation of the two monomers might appear, which could lead to variation in material properties during 3D printing. Therefore, miscibility studies have been performed. The components have been mixed as described above. Samples have been taken from the top of the mixture every hour for up to 8 hours and have been analyzed by Fourier-transform infrared (FTIR).

Furthermore, tensile testing of flat polymer test bars was performed in order to elucidate Young’s modulus for a later cell adhesion and colonization. Polymer test bars were prepared as S2 test bars following DIN 53504 and were measured based on DIN EN ISO 527-1.^15,16^

Soxhlet extraction was performed on 3 g polymer samples from each resin using 150 mL ethanol over a time period of 8 h followed by a subsequent 24 h period of drying under vacuum at room temperature. Polymer samples have been weighed and analyzed by FTIR spectroscopy before and after the extraction.

### Stereolithographic apparatus

**Figure 1.**
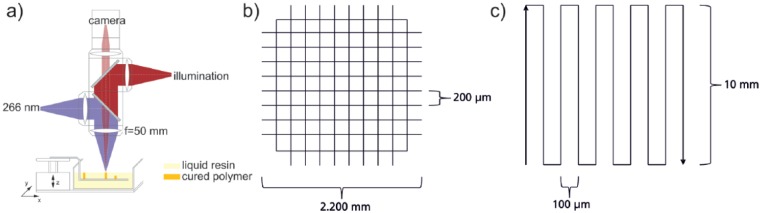
(a) Scheme of the used stereolithographic apparatus on the left and (b) illumination pattern for scaffold production (c)Illumination pattern for the meander-shaped 2.5D specimen.

A stereolithographic apparatus with resin A was used to build up a scaffold-like structure (Figure 1(a)). The used laser beam source is a pulsed frequency quadrupled Nd-YAG source (pulse t = 600 ns, λ = 266 nm, Horus Laser S.A.S, Limoges, France), which is focused on the surface of the resin. A platform is mounted on a z-axis and located in the vat. Z-position can be changed by moving this axis and x- and y-positions are controlled by the x- and y-table on which vat and axis are positioned.

For the 3D printing process, a working curve has to be determined by curing single-volume pixels (voxels) at various process parameters to adjust the layer thickness Δz to the curing depth c_d_.

Finally, the 3D scaffold was polymerized using a repetition rate f = 10 kHz, a feed rate of v = 5 mm s^−1^, a layer thickness Δz = 60 µm, a spot size w = 10 µm and a power P = 4.7 mW. Through repeated irradiation of a line pattern (Figure 1(a)), a scaffold structure consisting of 12 layers was structured.

The simple 2.5D-scaffold for functionalization experiments was produced with a similar stereolithographic setup. We used a laser beam source as a continuous frequency quadrupled Nd-YAG source (λ = 266 nm; TOPTICA Photonics, Gräfelfing, Germany), which is focused on the surface of the resin. Z-positioning is similar to the upper setup, while the beam is deflected in x- and y-direction using an optical scanner using a telecentric F-Theta lens (f = 53.5 mm). The used light power was P = 23 mW, the spot size w = 10 µm, and the feed rate 35 mm s^−1^. Irradiation of the meander-shaped pattern (Figure 1(c)) was repeated 200 times (Figure 1(c)).

### Cell isolation and culture

For experiments, human dermal fibroblasts (HDFs) were isolated from dermal biopsies approved by local ethics committee. The isolation was performed according to established protocols as follows: skin tissue was washed in 70% ethanol and thrice in phosphate-buffered saline (PBS, with 1% antibiotics/antimycotics (ABM)) followed by incubation in 50 IU/mL dispase solution (Gibco by Life Science) overnight at 4°C. To remove the epidermis from the dermal tissue, the skin was additionally incubated in dispase solution for up to 120 min at 37°C and 5% CO_2_ in humidified atmosphere at the next day. The dermis was minced with a scalpel and incubated in 100 IU/mL collagenase I (Gibco by Life Technologies) for 1 h at 37°C and 5% CO_2_. Occasionally, the cell solution was vortexed several times. Collagenase was neutralized by adding Dulbecco’s Modified Eagle’s Medium (DMEM, +10% fetal calf serum (FCS); Gibco Life Technologies), and cell-collagenase solution was strained and centrifuged at 500 *g* for 5 min. The remaining cell pellet was suspended in DMEM medium and seeded into cell culture flasks. HDFs were cultivated at 37°C and 5% CO_2_ up to passage 6.

### XTT cell proliferation assay with HDFs

XTT cell proliferation assay (Roche, Germany) was performed for the above-mentioned samples following ISO 10993-5 and ISO 10993-12.^17,18^ Specimens were washed with PBS, sterilized afterwards with 70% ethanol, and were allowed to dry under the sterile bench for several hours. Additionally, the materials were washed thoroughly in sterile Millipore water. Specimens were then extracted in DMEM (+10 % FCS) for 72 h (3 cm^2^/mL medium) at 37°C and 5% CO_2_ in a humidified atmosphere. In parallel, HDFs were seeded in 96-well plates with a final concentration of 5 × 10^3^ each well. The first 24 h, the cells were cultivated in DMEM medium without specimen eluates at 37°C and 5% CO_2_, followed by medium exchange with extracted medium. Cells were cultivated with the eluates up to 5 days and the assay was performed after 24, 72, and 120 h incubation time. Here, the XTT mixture was prepared by adding 20 µL of electron coupling reagent to 1 mL XTT labeling reagent; and 50 µL of the mixture was added to each well. Absorbance was measured right after adding the mixture (t = 0) and after 1, 2, 3, and 4 h at 475 nm with a reference wavelength of 630 nm using a luminescence reader (Tecan Infinite M200).^19^ For control, following conditions were chosen according to the ISO standards: cells cultivated in endothelial cell growth medium-2 (EGM-2; +10% FCS), EGM-2 (+10% FCS) with polyethylene (PE) tube (Braun, Germany) as negative control, and EGM-2 (+ 10 % FCS) with latex (Semperit, Germany) as positive control (Table 2).

**Table 2.** Overview of biocompatibility study.

EGM-2 (×10% FCS)	EGM-2 (×10% FCS) + PE tube	EGM-2 (×10 % FCS) + latex	EGM-2 (×10 % FCS + eluate of specimen A	EGM-2 (×10% FCS) + eluate of specimen B
Blank with cells (n = 3)	Positive control with cells (n = 3)	Negative control with cells (n = 3)	Eluate 1 with cells (n = 3)	Eluate 2 with cells (n = 3)
Blank without cells (n = 3)	Positive control without cells (n = 3)	Negative control without cells (n = 3)	Eluate 1 without cells (n = 3)	Eluate 2 without cells (n = 3)

EGM-2: endothelial cell growth medium-2; FCS: fetal calf serum; PE: polyethylene.

MTT test was done on multiwell plates choosing positive and negative controls, and eluate 1 and eluate 2 specimens with and without cells (n = 3).

Cell viability was determined by setting the absorbance of the EGM-2 control to 100% and adjusting the absorbance of the eluates in correlation to the 100%. Statistical analysis was performed using a one-way analysis of variance (ANOVA) with Tukey’s post hoc tests using SPSS software. A value of p < 0.05 was considered statistically significant.

### Functionalization of specimens

#### Propargyl acrylate as a (photo-)linker

For functionalization experiments, flat specimens on microscopic slides were prepared using off-stoichiometric resin B containing free thiol groups at the surface. Subsequently, the specimens were placed in crystallization glasses for surface functionalization and covered with an aqueous solution of propargyl acrylate in two different concentrations (Alfa Aesar, Ward Hill, MA, USA; 20 mL, 5 vol.% or 20 mL, 10 vol.%). The irradiation was done in a distance of 3 cm under the UV lamp for 12 h. Afterwards, the specimen was washed thoroughly with aqua dest. under slightly shaking conditions (5 times, 10 min). Successful surface functionalization with the linker was analyzed by ATR-FTIR-spectroscopy (Frontier; Perkin Elmer, Waltham, MA, USA) and fluorescent staining.

#### Functionalization with fluorescence dye

**Figure 2.**
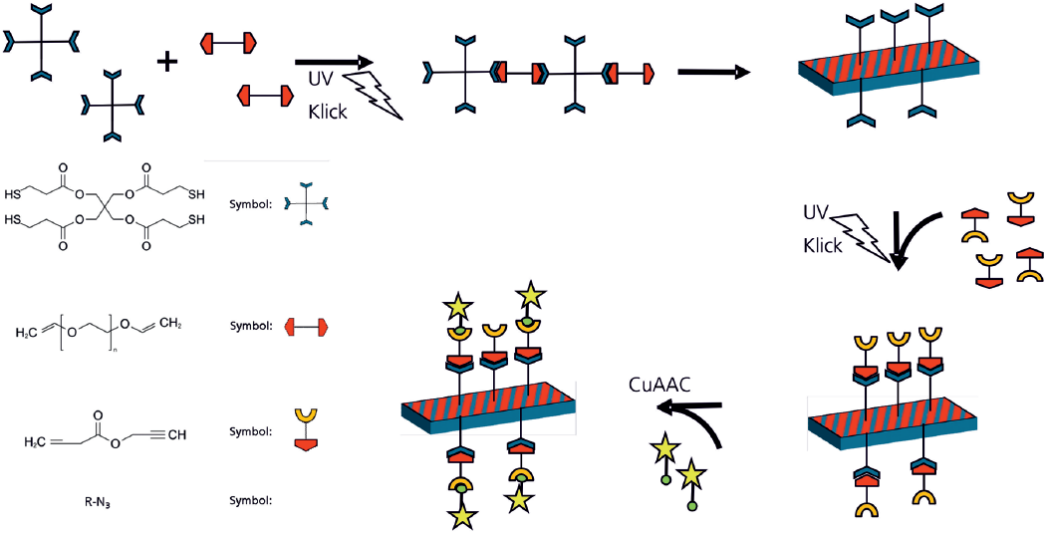
Concept of thiol-ene polymerization and surface functionalization. (Symbols: blue cross: pentaerythritol tetrakis(3-mercaptopropionate), red linker: poly(ethylene glycol) divinyl ether, red/yellow linker: propargyl acrylate, and green/yellow star: fluorescent dye containing azide.)

To prove chemical activity of the alkyne terminus of the linker propargyl acrylate, free alkyne groups from propargyl acrylate have been stained by copper-catalyzed click reaction using an azide functionalized fluorescence dye Alexa Fluor™ 488 Azide (Thermo Fisher Scientific). A dye solution (0.5 mg), dimethyl sulfoxide (DMSO; 100 µL), aqua dest. (1 mL), sodium ascorbate (26.6 mg) and copper (II) sulfate pentahydrate (13.3 mg) were prepared and the specimen surface was covered with this solution for 12 h at room temperature in the dark. Afterwards, the specimen was washed thoroughly with aqua dest. (5 times, 10 min); fluorescence microscopy (Olympus IX81; excitation 495 nm and emission 519 nm) was used (Figure 2).

## Results and discussion

### Preparation of flat specimens

Before UV-induced polymerization, the miscibility and stability of the mixture was tested. FTIR studies show that no change in the monomer composition occurs within 8 h, which complies with a useful processing time (supporting information). The polymerization reactions show different curing times of approximately 5 min for resin A and 10 min for resin B.

**Figure 3.**
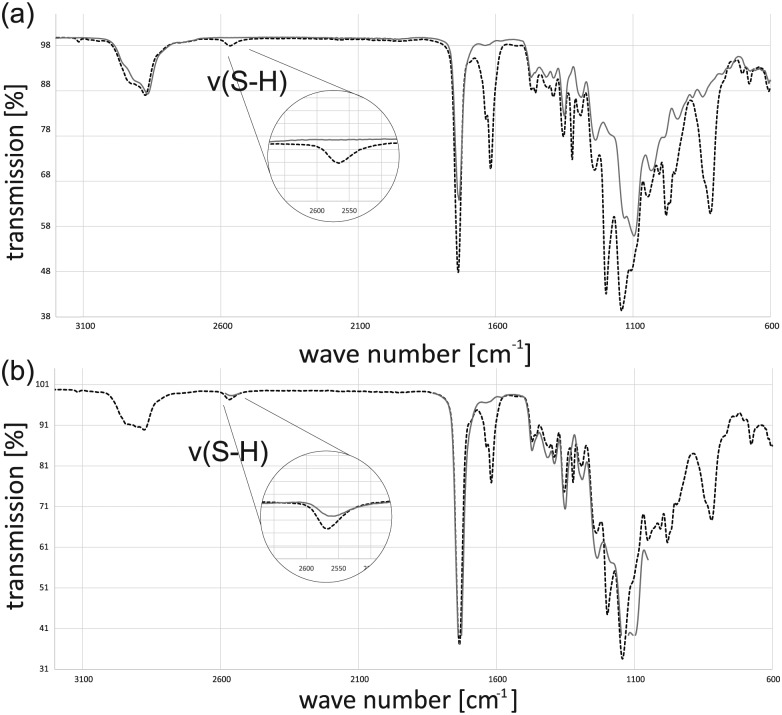
(a) FTIR spectra of resin A (black dotted line) and cured resin A (gray line) and (b) FTIR spectra of resin B (black dotted line) and cured resin B (gray line).

Both specimens appear to be colorless and clear with a smooth surface. To validate the polymerization process, FTIR analysis was done for the resins and the cured polymers. The FTIR spectra of the two uncured resins show the presence of the functional groups. The weak S-H stretch signal of the thiol groups can be found at around 2570 cm^−1^. The stronger C = C stretch of the vinyl groups can be found at 1620 cm^−1^. Comparing the spectrum of resin A with that of the cured resin A shows a strong decrease in the thiol- and vinyl-signal after the reaction (Figure 3). The vast majority of functional groups reacted with each other.

Doing the same experiment with the off-stoichiometric resin B also shows that almost all vinyl groups react but in contrast to resin A, there is still a signal for the S-H-stretch band. These signals are an indication for the presence of free thiol groups on the surface, but it is still not clear whether these groups can be used for further chemical surface treatments. The mechanical analysis of polymer test bars revealed a difference depending on the relative composition of the photoresins as reported by Carlborg et al.^14^ Polymers from resin A exhibit 6.9 ± 1.8 MPa, whereas polymers from resin B show 1.2 ± 0.5 MPa for Young’s modulus. As known from several works on soft tissue engineering, those moduli correspond to mechanical properties of blood vessels or heart valves.

Investigation by soxhlet extraction gave the following results: polymer samples from resin A show an increase in mass of 0.17%. In contrast, polymer samples from resin B demonstrate a mass reduction of 13.31%. FTIR spectroscopy (supporting information) confirms the findings from mass changes showing a decrease in aliphatic moieties, which might be attributed to the extraction of not fully crosslinked monomer or oligomer species.

### Biocompatibility with HFF cells

The evaluation of the cell proliferation assay showed the comparison of the control (EGM-2, +10% FCS) to the specimen eluates (specimens A and B). Here, data for the negative (PE tube) and positive control (latex) were not shown. The proliferation was observed over a period of 5 days; measurements were performed right after adding the XTT reagent 1, 2, 3, and 4 h after addition. For evaluation, only the measurement after 4 h of incubation was shown.

It was observed that control specimens showed a significantly higher proliferation rate compared to specimen 2. On day 1, an absorbance of 1.3 was detected for the EGM-2 control, while specimen A and specimen B showed significantly decreased absorbance values and thus lower proliferation signal (specimen A: 0.94, p value 0.033; sample B: 0.19, p value <0.000). Day 3 showed same tendency regarding proliferative behavior of cells. For the control specimens, an absorbance of 2.4 was measured. In contrast, specimen A showed a significantly lower absorbance (1.95, p value 0.021) than the control; moreover, the specimen B eluate seemed to have an enhanced inhibitory effect on the HDFs (0.06, p value < 0.000). On day 5, the proliferation in specimen A and the control had a non-significant difference (2.33 and 2.22). Whereas direct comparison of control and specimen B elucidated the cytotoxic effect of specimen B on the cell proliferation (0.53, p value < 0.000).

**Figure 4.**
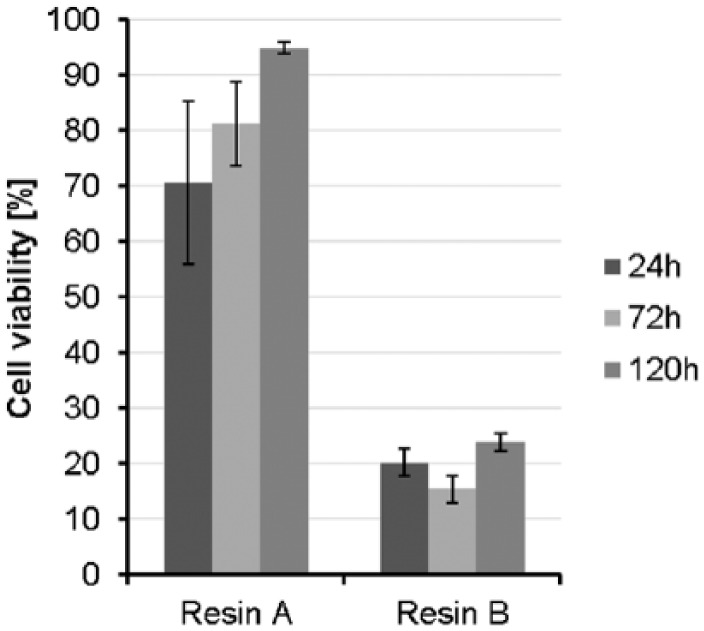
Viability of cells was tested after 24, 72, and 120 h. All results are normalized to the control group of the respective day.

For the percentage application of cell viability, the absorbance of the control specimens was set to 100% and values for eluates were adjusted to this baseline (Figure 4). According to the ISO standards, a cell viability of <70% reveals a cytotoxicity effect of materials on cells. Here, we observed a cell viability of 70.7% for specimen A and 20.3% for specimen B after incubating the eluates on cells for 24 h. Further investigations of day 3 (72 h) and day 5 (120 h) revealed increased cell viability for specimen A (81.25% on day 3; 94.95% on day 5) and specimen B (15.5% on day 3; 23.92% on day 5).

### 3D printing of thiol-ene scaffold structure

**Figure 5.**
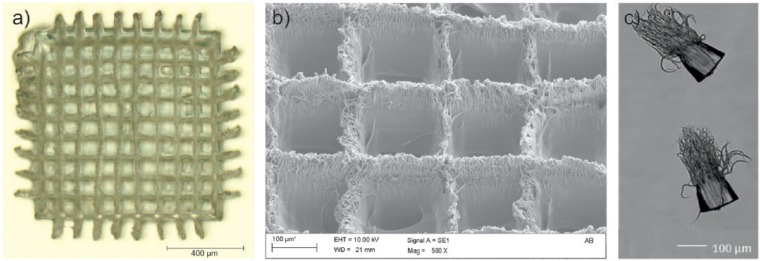
(a) Left: light microscope picture of the scaffold-like structure, (b) middle: SEM picture close-up of the structure pores and bars, and (c) right: SEM picture of single-volume pixels.

Using the stoichiometric resin A, a scaffold-like structure was printed with the given parameters. A light microscopy picture of this structure shows that the design of the template is similar to that of the irradiated and polymerized pattern (Figure 5(a)). The bars have a thickness between 20 and 40 µm and the rectangle pores have a width of 110–130 µm. The central rectangle pores are smaller than the ones in the periphery of the structure. In addition, bars in the center are not perpendicular to each other anymore. The reason for this may be volume shrinkage during the polymerization and therefore polymerization stress in the printed structure. Although thiol-ene polymers have a delayed gelation and therefore less polymerization stress than comparable, (meth-)acrylic polymer^9^ thiol-ene networks still undergo shrinkage, especially for crosslinked polymers as used in this work. Although it cannot be ruled out that the deformation of the structure appeared during the process or during post processing. Hence, further studies have to be done to clarify the origin of these deformations.

A scanning electron microscopy picture of the structure shows that the surface of the structure has a high roughness (Figure 5(b)). A filament structure is observed on the surface. In some pores, these filaments even cross a pore. From the voxel tests for determining the working curve, it is known that these filament structures occasionally appear on the bottom side of these polymers during the laser curing process (Figure 5(c)).

The origins of these filament structures are still unknown, but it can be assumed that self-focusing may be a reason for this effect.^20,21^ Photopolymerization-induced refractive index variation is a well-known effect.^22–24^ Thus, the liquid and later the polymer works as an optical lens during the irradiation. In addition, optical bleaching of the resin during the polymerization could also be an explanation for the generation of filaments.^25^ During the irradiation of a photoinitiator, the thiol component is transferred to an excited singlet and triplet state, which may change the optical properties of the molecule drastically. Especially for high-irradiation intensities, this effect can lead to locally uncontrolled voxel growth.

### Functionalization of specimens

#### Propargyl acrylate as a photolinker

In previous experiments, we demonstrated that surfaces of the materials are cell repellent, but coating with poly-L-lysin allows cell cultivation (unpublished data). This leads us to the controlled functionalization of polymer surface using off-stoichiometric resins with an excess of thiol groups. In this study, we used a high thiol to alkene ratio of 2:1 to make sure that a huge amount of thiol groups is on the surface and can be detected by FTIR as by fluorescence staining later on. The FTIR analysis confirmed the presence of free thiol groups in the B-polymer, but it is unknown whether the groups are just on the surface or even in volume of the polymer and therefore sterically hindered for further reactions. For this reason, we tried to couple reactive propargyl acrylate groups to the thiol groups which can again react photochemically via thiol-ene click chemistry. Propargyl acrylate can react with both functional groups, the terminal alkyne and the alkene group, but it is known that alkene groups react much faster under irradiation than alkynes.^26^ Afterwards, we stained these functional groups with a copper-catalyzed alkyne-azide click reaction.

**Figure 6.**
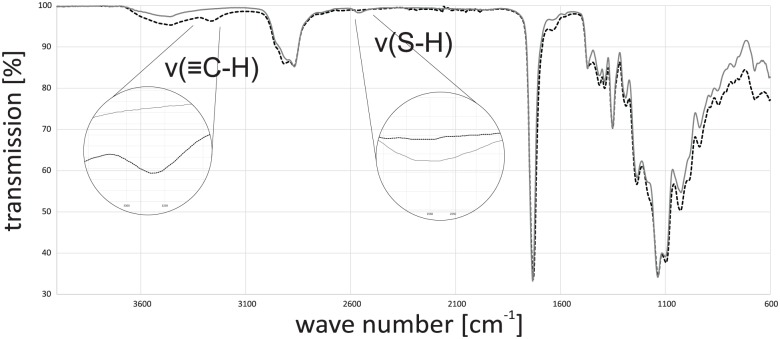
FTIR spectra of cured resin B (gray line) and cured resin B functionalized with linker propargyl acrylate (black dotted line).

Incubation of the polymer surface with the pure linker leads to cracking of the specimen surface because the liquid linker diffuses into the specimen and the highly crosslinked polymer cracks during swelling. Thus, an aqueous solution of propargyl acrylate and a long reaction time was used for the functionalization. A specimen made from off-stoichiometric resin B was functionalized with the linker and thoroughly washed. A comparison of the FTIR spectra of the two specimens shows that the functionalization was successfully achieved (Figure 6). The S-H stretch band, which was clearly observable, is disappeared and in addition for the functionalized specimen, an alkyne-C-H peak at 3270 cm^−1^ appeared. Control tests with addition of the linker and without irradiation confirm the results of successful linkage. Neither an alkyne signal has appeared nor did the thiol-signal disappeared. Hence, functionalization on a specific local position could be achieved by local irradiation.

#### Printing of scaffolds and subsequent fluorescence staining

**Figure 7.**
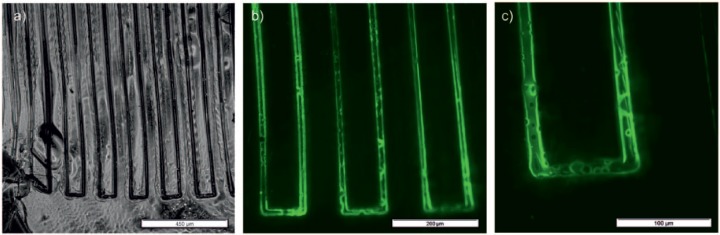
(a) Light microscopy image of stained 2.5D specimen, (b) fluorescence microscopy image of stained 2.5D specimen 10×, and (c) fluorescence microscopy image of stained 2.5D specimen 20×.

In a subsequent step to the FTIR analysis, a visualization of successful surface functionalization on 2.5D specimens was done by staining the alkyne groups with a fluorescent dye. Light microscopy imaging shows the meander-shaped structure of the stained 2.5D specimen (Figure 7(a)). The track pitch of the printed straight lines with length of 10 mm is about 100 µm, but it can be seen that the lines are not straight due to polymerization stress or detachment of polymer from the microscopy slide. Fluorescence microscopy imaging in different magnification of 10× and 20× (Figure 7(a)–(c)) shows bright fluorescence signals on the surface of the polymeric specimen and less brightness for the surrounding fused silica surface. The highest fluorescence intensities are observed at the slopes of the printed structures, which is most probably related to the observing angle of the fluorescence microscopy. The results show that the attached linker is chemically active and a functionalization at the surface of a laser-polymerized structure is possible.

## Conclusion

Thiol-ene-based photoresins are a promising material for the use in 3D bioprinting. In addition to the known positive aspects for bioprinting approaches, thiol-ene photoresins also offer the possibility of initiator-free 3D printing with subsequent surface functionalization.

For the combination of poly(ethylene glycol) divinyl ether and pentaerythritol tetrakis(3-mercaptopropionate), we could demonstrate that polymerization of a stoichiometric amount of monomers lead to a biocompatible polymer and that a 2:1 excess of thiol groups in the monomer solution leads to functional thiol groups on the surface of the cured structure. These groups can be used for surface functionalization, for example, with a linker molecule such as propargyl acrylate. Unfortunately, this ratio of monomers leads to polymers with high cytotoxicity within cytocompatibility testing. Perspectively, lower ratios of excess thiol groups between 1.1:1 and 1.5:1 have to be tested to improve the cytocompatibility while maintaining the activity of surface functional groups. As shown, the monomer ratio directly influences the mechanical properties of the resulting polymer. In the future, this offers the possibility of designing material properties for adhesion of a specific cell type. Soxhlet extraction experiments showed an intense mass decrease for the off-stoichiometric specimens. Hence, improving washing procedures have to be elaborated to remove remaining monomers from the bulk to increase the cytocompatibility. A bioactive surface functionalization may increase cytocompatibility as well. Additionally, long-term stability and long biocompatibility testing will be necessary to evaluate the suitability of the given approach for 3D bioprinting.
